# Pre-contoured superior locking plates offer poor bone fit for midshaft clavicle fracture fixation: cadaveric analysis of 4 commercially available systems

**DOI:** 10.1590/0100-6991e-20223177en

**Published:** 2022-04-13

**Authors:** ADRIANO FERNANDO MENDES, AUGUSTO KHEDE TAVARES, IGOR GERDI OPPE, ROBINSON ESTEVES PIRES, WILLIAM DIAS BELANGERO, PEDRO JOSÉ LABRONICI

**Affiliations:** 1 - Hospital Universitário da Universidade Federal de Juiz de Fora, Serviço de Ortopedia e Traumatologia - Juiz de Fora - MG - Brasil; 2 - Hospital Santa Teresa, Serviço de Ortopedia e Traumatologia - Petropolis - RJ - Brasil; 3 - Universidade Federal de Minas Gerais, Faculdade de Medicina - Belo Horizonte - MG - Brasil; 4 - Universidade Estadual de Campinas, Faculdade de Medicina - Campinas - SP - Brasil; 5 - Universidade Federal Fluminense, Faculdade de Medicina - Niterói - RJ - Brasil

**Keywords:** Bone Plates, Clavicle, Fracture Fixation, Internal, Placas Ósseas, Clavícula, Fixação de Fratura, Interna

## Abstract

**Objective:**

The goal of this study is to verify how commercially available pre-contoured superior plates fit on clavicle midshaft fractures.

**Methods:**

100 cadaveric clavicles were evaluated by three distinct observers applying the clavicle congruence score and comparing four different 6 to 8-hole pre-contoured anatomic locking-plate systems.

**Results:**

the inter-observer agreement was considered moderate by the percentage agreement and fair by the Fleiss’ Kappa, with no significant differences between evaluations. Only 1 of the 8 plates presented an anatomic fit greater than 70%. Long plates (8 holes) presented a poor fit compared to short plates (6 or 7 holes).

**Conclusions:**

the overall evaluation showed that currently-available pre-contoured superior plate systems provide a poor fit on clavicles for midshaft fracture fixations. Long plates present a worse fit compared to short ones.

## RESUMO

**Objetivo:**

verificar como as placas superiores pré-moldadas disponíveis no mercado se adaptam às fraturas do terço médio da clavícula.

**Método:**

100 clavículas de cadáveres foram avaliadas por três observadores distintos, aplicando-se o escore de congruência da clavícula e comparando quatro sistemas diferentes de placas bloqueadas anatômicas pré-moldadas de seis a oito furos.

**Resultados:**

a concordância interobservador foi considerada moderada pelo percentual de concordância e regular pelo índice Kappa de Fleiss, sem diferenças significativas entre as avaliações. Apenas uma das oito placas apresentou encaixe anatômico maior que 70%. As placas longas (oito furos) apresentaram um encaixe ruim em comparação com as placas curtas (seis ou sete furos).

**Conclusões:**

os sistemas de placa superior pré-moldadas disponíveis atualmente fornecem um encaixe inadequado às clavículas para fixações de fraturas do terço médio. Placas longas apresentam um encaixe pior que as curtas.

## INTRODUCTION

Clavicle fractures correspond to about 4% of all adult fractures, with an annual incidence, in Sweden, of 50/100.000 cases^1^. Approximately 80% occur in the midshaft^2^. The ideal treatment for displaced diaphyseal clavicle fractures is unknown^3^, but those in favor of surgical management argue that it is associated with a lower risk of nonunion and better functional outcomes^4^. For this choice, the most popular surgical technique is open reduction and internal fixation using plate and screws^5^. However, a high implant removal rate is also described^3^.

Pre-contoured locking plates were developed to fit perfectly on the shape of the clavicle, reducing surgical time, causing less soft-tissue discomfort, and increasing fixation stability due to proper bone-implant contact^6^ ^,^ ^7^. However, the clavicle presents a unique and complex anatomy^8^ with different gender and ethnic characteristics^6^ ^,^ ^9^ ^,^ ^10^, thereby precluding the pre-molded implant from perfectly fitting the bone shape^6^, which might lead to clinical issues such as reduction failure and post-operative soft-tissue discomfort. In a Randomized Clinical Trial with clavicle fractures treated surgically using superior pre-countered plates, the hardware-removal rate was of 53%^11^. 

Superior clavicle-plate adaptability was first described by Huang^6^. In an attempt to clarify the adaptability of pre-countered plates on the superior surface of the clavicle, Malhas et al.^12^ developed a plate congruence score. This study aims at comparing four pre-contoured superior locking-plate systems, with different plate sizes for midshaft clavicle fractures in terms of bone-surface accommodation, using the clavicle congruence score. The hypothesis is that there are plate fit differences according to the size and system brand 

## METHOD

This research project was evaluated and approved by the institution’s research and ethics committee (protocol: 80469417.0.0000.5245). This study used one hundred anatomical clavicles from skeletally mature individuals not identified by sex or race with the adjacent soft parts completely disinserted and showing no previous signs of fractures. The clavicles were numerically and sequentially cataloged from 1 to 100 and organized into ten sets of ten specimens, following the numerical sequence with no randomization of the specimens. The upper and lower surfaces and the medial and lateral extremities were identified on all clavicles. The length of each clavicle was measured using an analog caliper (FORTGPRO^®^ - model FG8330) and its midpoint was identified. After identifying the surfaces, the clavicles were separated into left and right-side groups for sampling purposes. In all, 52 right clavicles and 48 left clavicles were separated for evaluation.

Implants from four different manufacturers - Arthrex^®^ (Naples, FL, USA), Johnson & Johnson^®^/DePuySynthes^®^ (Warsaw, IN, USA), Kanghui^®^/Medtronic^®^ (Changzhou, JS, China), Hexagon^®^ (Itapira, SP, Brazil) - were used to check how each implant fit in a hypothetical diaphyseal clavicle fracture fixation. In order to guarantee the comparison between manufacturers, the short (six or seven holes) and long (eight holes) implants were selected from each company. 

The degree of accommodation of the implant was evaluated according to the clavicle congruence score (CCS), described by Malhas et al.^12^ Graded from one to three, where a rating of three (3) represents an anatomic fit, with the plate perfectly adapted to the bone, two (2) represents a good fit, with anterior or posterior plate protrusion, but with each plate hole centralized on the bone, and one (1) represents a poor fit, with a complete discrepancy between the plate and the clavicle, with one or more plate holes without screws on the bone.

**Figures 1A and 1B:**
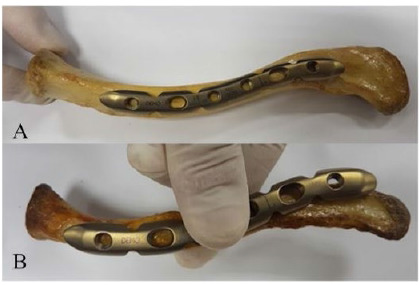
Anatomic and poor degree of CCS.

Figures 1A and 1B demonstrate the CCS for the same implant, in anatomic and poor fits, respectively.

Each examiner evaluated all clavicles on the same day for a single plate sequentially by sets of ten specimens. The fit of the eight plates was verified for each clavicle. The examiner was allowed to start the evaluation of the next plate in the subsequent day. The adequacy of the implants was checked freehand, without the use of reduction clamps, to avoid damaging the anatomical specimens. Each plate was placed individually in the upper position, with the correct correspondence of the medial and lateral edges of the implant, according to the manufacturer’s description. Starting with the first plate, the examiner assessed the CCS one time for the first clavicle; then, he repeated this two more times for this same clavicle, totaling three assessments with this implant for that clavicle. Next, the same plate was maintained, used again and the second clavicle was evaluated. Therefore, for each plate, the examiner performed 300 evaluations. Since there were eight implants, a total of 2400 evaluations were performed per examiner. 

The midpoint longitudinal mark simulated a transverse fracture, and the short implants were positioned to ensure three free holes on each side of the plate. The multifragmented fracture pattern was estimated when selecting the long implant to be evaluated. The length of the multifragmented fracture line was not predetermined, so it was presumed that the use of a longer implant would be necessary for this fracture pattern. In these cases, the examiner was instructed not to consider the two center plate holes and position the three holes on either side in the most distant portions of the midpoint.

The evaluations were carried out by three different examiners with varying degrees of experience: an orthopedic surgeon (evaluator 1) with more than eight years of surgical experience in orthopedic trauma; two medical residents in an orthopedic-surgery post-graduation program: one in the third year (evaluator 2), and another in the first (examiner 3). 

The descriptive analysis of the data aimed at recording the distribution of the clavicle length measurements and analyzing the CCS frequency. The inferential analysis aimed at evaluating the statistical significance of the differences observed between the left and right clavicle measurements and the significance of the differences found in the CCS frequency distributions for the clavicles of different subgroups. The hypothesis of normality in the clavicle length distribution was verified by the Kolmogorov-Smirnov (KS) tests and the Shapiro-Wilk test (SW). The difference-significance analysis found in the CCS frequency distributions for different subgroups was analyzed by the Chi-square test. The intra and inter-observer agreement was assessed by the percentage agreement and the Fleiss’ Kappa, both interpreted as follows [13]: values ≤0 indicated no agreement, 0.01-0.20 no to slight agreement, 0.21-0.40 fair agreement, 0.41-0.60 moderate agreement, 0.61-0.80 substantial agreement, and 0.81-1.00 an almost perfect agreement. All statistical tests were 2-sided, with a p-value set to 0.05. The data were analyzed using statistical analysis software R, version 3.1.0, and with the IBM SPSS program (Statistical Package for the Social Sciences), version 22.0.

## RESULTS

The clavicles measured 11.50 to 17.5cm, with an average length of 14.27cm (SD=1.16) (Table 1). The variability of the sample was very low. The percentiles showed that only 5% of the clavicles were less than 12.50cm long, and only 5% were over 16.00cm: 90% measured between 12.50 and 16.00cm. Clavicle length measurements followed a normal distribution, given the normality test p-values, both overall and in the right- and left-side clavicle subgroups. Levene’s test results did not show any significant difference between clavicle measurements in both groups (p-value=0.776). There was no significant difference between right and left mean sizes (p-value=0.329), according to the Student’s t-test.

**Table 1:** The main statistics on the lengths of the clavicles evaluated, by side and overall.

Statistic	Right Side	Left Side	Overall
Minimum	11.50	12.00	11.50
Maximum	17.50	17.50	17.50
Mean	14.16	14.39	14.27
Median	14.15	14.50	14.30
5^th^ percentile	12.40	12.60	12.50
25^th^ percentile	13.20	13.55	13.50
75^th^ percentile	15.00	15.10	15.00
95^th^ percentile	15.70	16.00	16.00
SD^a^	1.19	1.12	1.16
CV^b^	0.08	0.08	0.08
KS^c^ (p-value)	0.200	0.200	0.200
SW^d^ (p-value)	0.639	0.662	0.309
Levene (p-value)	-	0.776	-
Student's t (p-value)	-	0.329	-

Notas: ^a^SD: Standard Deviation; ^b^CV: Coefficient of Variability; ^c^KS: Kolmogorov-Smirnov test; ^d^SW: Shapiro-Wilk test.

We classified the sample by length, and according to the 25^th^ and 75^th^ distribution percentile values, they were categorized into small, regular, and large. A clavicle was considered small (24 specimens) if its length was less than 14cm (25^th^ percentile); regular (58 specimens) if its length was greater than or equal to the 25^th^ percentile and less than or equal to the 75^th^ percentile; and large (18 specimens) if its size was greater than 15cm (75^th^ percentile).

In the percentage agreement evaluation, the intra-observer analysis showed that evaluator 1 agreed in 0.68 (SD=0.08) of the cases (substantial agreement); evaluator 2 agreed in 0.71 (SD=0.10) (substantial agreement), and evaluator 3 agreed in 0.81 (SD=0.05) (almost perfect agreement). In the homogeneity analysis, all showed low variability. The inter-observer results for the three evaluators were interpreted as moderate agreement [evaluator 1=0.45 (SD=0.135), evaluator 2=0.41 (SD=0.13), and evaluator 3=0.41 (SD=0.11)], with no significant difference between the evaluations.

In the analysis according to the Fleiss’ Kappa, the intra-observer analysis showed that evaluator 1 had “substantial” agreement (Kappa=0.63; 95% CI 0.60-0.66), evaluator 2 had “moderate” agreement (Kappa=0.60; 95% CI=0.57-0.63), and evaluator 3 had “substantial” agreement, (Kappa=0.76; 95% CI=0.71-0.81). In the inter-observer analysis, the agreement between the three evaluators was ‘fair’, with the following values per evaluator: evaluator 1: Kappa=0.27; 95% CI=0.24-0.30; evaluator 2: Kappa=0.24; 95% CI=0.21-0.27; evaluator 3: Kappa=0.25; 95% CI=0.22-0.28. There were no significant differences between evaluations.

Table 2 shows the distribution per plate, per evaluator, and overall. The results were different between the evaluators. For evaluators 1 and 2, the plate with the lowest “Poor Fit” percentage was the Kanghui^®^/Medtronic^®^ (KG) 6-hole plate, and for evaluator 3 it was the Johnson&Johnson^®^/DePuy Synthes^®^ (J&J) 6-hole plate. For evaluator 1, the highest “Anatomic Fit” percentage rating was found for the KG 6-hole plate. For evaluators 2 and 3, the highest “Anatomic Fit” ratings were found for the J&J 6-hole plate. In the overall analysis, the Arthrex 7-hole plate had the lowest “Poor Fit” percentage and the J&J large 8-hole plate had the highest “Poor Fit” percentage classification. The J&J short 6-hole plate had the highest “Anatomic Fit” percentage classification. The Arthrex large 8-hole and Hexagon 8-hole plates had the lowest “Anatomic Fit” percentages.

**Table 2:** Fit data, according to CCS, by plate, evaluator and overall rating, highlighted the lowest and the highest CCS.

		Evaluator 1		Evaluator 2		Evaluator 3		Overall	
Plate	CCS	n	%	n	%	n	%	n	%
Arthrex 7	Poor	14	4.7	10	3.3	9	3.0	33	3.7
	Good	99	33.0	81	27.0	80	26.7	260	28.9
	Anatomic	187	62.3	209	69.7	211	70.3	607	67.4
		Evaluator 1		Evaluator 2		Evaluator 3		Overall	
Plate	CCS	n	%	n	%	n	%	n	%
Arthrex 8	Poor	61	20.3	72	24.0	74	24.7	207	23.0
	Good	99	33.0	86	28.7	78	26.0	263	29.2
	Anatomic	140	46.7	142	47.3	148	49.3	430	47.8
J&J^a^ 6	Poor	64	21.3	9	3.0	7	2.3	80	8.9
	Good	104	34.7	32	10.7	44	14.7	180	20.0
	Anatomic	132	44.0	259	86.3	249	83.0	640	71.1
J&J 8	Poor	159	53.0	42	14.0	65	21.7	266	29.6
	Good	80	26.7	55	18.3	73	24.3	208	23.1
	Anatomic	61	20.3	203	67.7	162	54.0	426	47.3
KG^b^ 6	Poor	3	1.0	5	1.7	78	26.0	86	9.6
	Good	68	22.7	83	27.7	76	25.3	227	25.2
	Anatomic	229	76.3	212	70.7	146	48.7	587	65.2
KG 8	Poor	106	35.3	76	25.3	63	21.0	245	27.2
	Good	94	31.3	126	42.0	109	36.3	329	36.6
	Anatomic	100	33.3	98	32.7	128	42.7	326	36.2
Hexagon 6	Poor	64	21.3	18	6.0	42	14.0	124	13.8
	Good	134	44.7	136	45.3	101	33.7	371	41.2
	Anatomic	102	34.0	146	48.7	157	52.3	405	45.0
Hexagon 8	Poor	57	19.0	18	6.0	62	20.7	137	15.2
	Good	138	46.0	134	44.7	106	35.3	378	42.0
	Anatomic	105	35.0	148	49.3	132	44.0	385	42.8

*Notes:* ^*a*^ *J&J: Johnson & Johnson* ^*®*^ */DePuy Synthes* ^*®*^ *.* ^*b*^ *KG: Kanghui* ^*®*^ */Medtronic* ^*®*^ *.*

**Figure 2:**
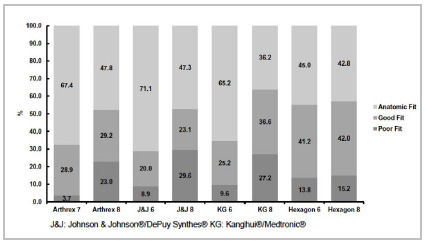
Distribution of frequencies of overall CCS of the eight different plates.

Figure 2 shows the fit classification frequencies without discriminating the evaluator, obtained for the different plate brands and models. When comparing the fit classification of plates of the same brand on a clavicle using the chi-square test, the p-value=0.000 was obtained for all classification comparisons (Arthrex 7 vs. Arthrex 8 holes; J&J 6 vs. J&J 8 holes; KG 6 vs. KG 8 holes; Hexagon 6 vs. Hexagon 8 holes), that is, the fit rating of a plate from the same brand was significantly associated with the size of the plate.

**Figure 3:**
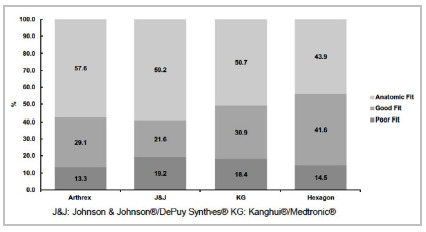
Distribution of overall rating of plate’s fit by different systems.

When comparing the frequencies of plates of the same brand, it was found that there was a significant difference between the poor, good, and anatomic CCS of small and large Arthrex (p-value=0.000), J&J (p-value=0.000), and KG (p-value=0.000) plates. There was no significant difference between the Hexagon plates (p-value=0.544). The fit classification of several brands without discriminating the size of the plate can be seen in Figure 3. Altogether, without discriminating the evaluator, Arthrex had the lowest (13.3%) and J&J had the highest “Anatomic fit” rating percentages (59.2%).

**Figure 4:**
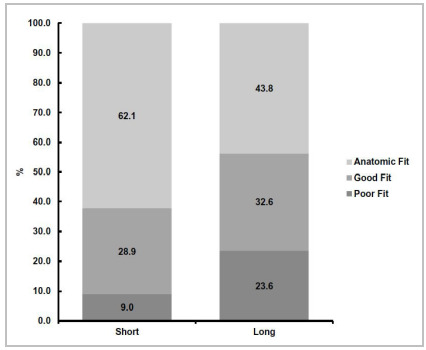
Differences in adaptation according to plate length.

Regarding the aggregated distribution data for short and long plates (Figure 4), it was observed that the CCS scores were significantly different and that the small plates had better anatomical fit performance than the large ones (p-value=0.000).

## DISCUSSION

In the present study, we evaluated the accommodation of 8 different clavicle plates according to the score from Malhas et al.^12^, and the results showed that the majority of implants did not present high anatomic fit levels, and that the long ones where deemed to have poor performance compared to short ones. This is relevant information since the surgeon must be aware that some plate adjustments will be needed to achieve proper fracture fixation. Careful preoperative planning with adequate imaging evaluation is recommended aiming at correctly understanding the patient anatomy, thereby saving surgical time. Bauer et al.^14^, analyzing the fit of a long (8-hole) clavicle plate, described that it was poor compared to a 3.5mm reconstruction plate. In spite of the higher fatigue resistance of anatomical implants in comparison to recon plates in a superior position^6^, even in the simulation of comminuted fractures^15^, a careful evaluation by the surgeon is advised when choosing an implant with a better fit and less resistance^14^ ^,^ ^16^ or a more resistant anatomical implant with lower fit probability^14^ ^,^ ^16^. 

The low profile pre-contoured locking plate is the most popular implant for displaced midshaft clavicle fracture fixation^6^ ^,^ ^12^, but instead of an anatomic accommodation to the shape of the clavicle, the data describe the poor performance of the studied implants^14^ ^,^ ^17^ ^,^ ^18^. Our results demonstrated that for all brands evaluated, the poor fit rate varied up to 19.2%. It is noteworthy that, in terms of anatomic characteristics and anthropometric measurements of the sample, our findings are quite similar to previous studies with different populations, demonstrating that even with many anatomic similarities, a low appropriate plate-accommodation level still persists^9^ ^,^ ^14^ ^,^ ^17^. 

One of the complications after surgical fixation of displaced clavicle fractures is the high implant removal rate. The advantage of the clavicle plate would be its ability to cause less soft tissue discomfort^6^, diminishing the need for plate removal. The results of our study show that, in many evaluations, a considerable portion of hardware lay beyond the bone. This may be associated with the persistence of soft tissue irritation and the need for implant removal, which is supported by the literature with the removal rate for pre-countered plates ranging from 12.7^18^ to 53%^11^.

The CCS might help surgeons in choosing the appropriate implant. Despite its reproducibility being questionable, especially due to the fair inter-observer agreement, our results are similar to other studies that replicated the method^9^. In a clinical perspective, the score can be used in preoperative planning with 3D-printed replicas of the fractured clavicle, increasing the ability to better assess the fit of the implants and reduce plate-related discomfort^19^.

The choice of two statistics for the evaluation of CCS among the three examiners with different levels of experience in orthopedic surgery is supported by the literature. According to McHugh, in situations where varying levels of training or guesswork are expected among observers, it is recommended to evaluate both the percentage agreement and the Kappa^13^.

Another matter deserves to be pointed out in the present study: to overcome a potential bias when comparing implants that were developed for a specific population, we compared four short and four long plates manufactured in North America, Asia, and Latin America. Nevertheless, only one implant (J&J) presented an anatomic classification in over 70% of the cases. Moreover, even observers with different knowledge levels in orthopedic surgery presented similar behaviors, in line with studies published about the inadequate fit of pre-contoured clavicle plates^9^ ^,^ ^12^ ^,^ ^17^.

A potential limitation of this study is that in the sample of 100 clavicles it was supposed that the ethnic characteristics were of a single population, even if they were not identified according to gender or race. Another limitation is that the previous sample size calculation according to the measurement of agreement was not performed because the consulting statistician considered that the number of evaluations per examiners was large enough for the pragmatic purpose of the study.

As a strength of our study, we believe that the method of evaluating the sample with different plates, different brands and examiners with different levels of experience simulated the surgical practice of choosing the implant and demonstrated that there is a difference in the fit of all implants.

Since the clavicle is a unique bone with its own singularities and a complex anatomy, designing an ideal implant remains a challenging obstacle. With the continuous implant-engineering development, future directions towards customized implants for clavicle fractures^20^ individualized according to the patient anatomy can be a promising alternative.

## CONCLUSION

The vast majority of implants tested presented less than 70% anatomic plate fit, according to the clavicle congruence score. Long implants showed a poor fit, compared to the short plates. These results point out that an ideal implant aiming at the anatomical fit on different clavicle shapes is unlikely. Future tendencies towards customized implants may be an effective alternative to decrease surgical time and minimize implant removal rates.
